# A third vaccine dose equalises the levels of effectiveness and immunogenicity of heterologous or homologous COVID-19 vaccine regimens, Lyon, France, December 2021 to March 2022

**DOI:** 10.2807/1560-7917.ES.2023.28.15.2200746

**Published:** 2023-04-13

**Authors:** Nicolas Guibert, Kylian Trepat, Bruno Pozzetto, Laurence Josset, Jean-Baptiste Fassier, Omran Allatif, Kahina Saker, Karen Brengel-Pesce, Thierry Walzer, Philippe Vanhems, Sophie Trouillet-Assant, on behalf of the Lyon-COVID study group

**Affiliations:** 1 Occupational Health and Medicine Department, Hospices Civils de Lyon, Université Claude Bernard Lyon1, Université Gustave Eiffel-IFSTTAR, UMRESTTE, UMR T_9405, Lyon University, Lyon, France; 2 CIRI - Centre International de Recherche en Infectiologie, Université de Lyon, Université Claude Bernard Lyon 1, Inserm, U1111, CNRS, UMR5308, ENS Lyon, Université Jean Monnet de Saint-Etienne, Lyon, France; 3 Joint Research Unit Civils Hospices of Lyon-bioMérieux, Hospices Civils de Lyon, Lyon Sud Hospital, Pierre-Bénite, France; 4 Laboratoire des Agents infectieux et d‘Hygiène, University Hospital of Saint-Etienne, Saint-Etienne, France; 5 Virology Laboratory, Institute of Infectious Agents, Laboratory Associated with the National Reference Centre for Respiratory Viruses, Hospices Civils de Lyon, Lyon, France.; 6 GenEPII sequencing platform, Institute of Infectious Agents, Hospices Civils de Lyon, Lyon, France; 7 Service D'Hygiène, Épidémiologie, Infectiovigilance et Prévention, Hôpital Édouard Herriot, Hospices Civils de Lyon, Lyon, France; 8 The members of the project group are acknowledged at the end of the article; * These authors contributed equally and share last authorship

**Keywords:** SARS-CoV-2, vaccination, heterologous vaccine regimens, breakthrough infection, healthcare workers, neutralisation

## Abstract

**Background:**

To cope with the persistence of the COVID-19 epidemic and the decrease in antibody levels following vaccination, a third dose of vaccine has been recommended in the general population. However, several vaccine regimens had been used initially for the primary vaccination course, and the heterologous Vaxzevria/Comirnaty regimen had shown better efficacy and immunogenicity than the homologous Comirnaty/Comirnaty regimen.

**Aim:**

We wanted to determine if this benefit was retained after a third dose of an mRNA vaccine.

**Methods:**

We combined an observational epidemiological study of SARS-CoV-2 infections among vaccinated healthcare workers at the University Hospital of Lyon, France, with a prospective cohort study to analyse immunological parameters before and after the third mRNA vaccine dose.

**Results:**

Following the second vaccine dose, heterologous vaccination regimens were more protective against infection than homologous regimens (adjusted hazard ratio (HR) = 1.88; 95% confidence interval (CI): 1.18–3.00; p = 0.008), but this was no longer the case after the third dose (adjusted HR = 0.86; 95% CI: 0.72–1.02; p = 0.082). Receptor-binding domain-specific IgG levels and serum neutralisation capacity against different SARS-CoV-2 variants were higher after the third dose than after the second dose in the homologous regimen group, but not in the heterologous group.

**Conclusion:**

The advantage conferred by heterologous vaccination was lost after the third dose in terms of both protection and immunogenicity. Immunological measurements 1 month after vaccination suggest that heterologous vaccination induces maximal immunity after the second dose, whereas the third dose is required to reach the same level in individuals with a homologous regimen.

**Key public health message**

**What did you want to address in this study?**

Different vaccine combinations were initially used to immunise populations against COVID-19. Others and we previously found that the mixed Vaxzevria/Comirnaty combination was more effective and immunogenic than the matched Comirnaty/Comirnaty one. We wanted to determine if this benefit was retained after the third vaccine dose.

**What have we learnt from this study?**

In a study of SARS-CoV-2 infections among vaccinated healthcare workers, we confirmed that mixed vaccination regimens were more protective against infection than matched regimens, but this was no longer the case after the third dose. Antibody titres and neutralising capacity were higher after the third dose than after the second dose in the matched regimen group, but not in the mixed group.

**What are the implications of your findings for public health?**

A third dose of mRNA vaccine significantly improves the antibody response against SARS-CoV-2, and mixed combinations of vaccines are not required to reach good immunity if a third dose is administered.

## Introduction

In response to the COVID-19 pandemic, several vaccines were rapidly designed and administered to the population, inducing a protective immunity composed of both neutralising antibodies and virus-specific T lymphocytes. As multiple vaccines were available, different heterologous combinations of prime/boost doses have been used in patients. This mixing of vaccines was motivated first by the necessary adaptation to limited vaccine supply, but also by the rare observation of vaccine-induced severe adverse reactions with some vaccines. 

Most studies have reported similar or higher immunogenicity following heterologous primary vaccination involving Vaxzevria (ChAdOx1nCoV-19, AstraZeneca, Cambridge, United Kingdom) and the mRNA vaccines Comirnaty (BNT162b2, BioNTechPfizer, Mainz, Germany/New York, United States (US)) and Spikevax (mRNA-1273, Moderna, Cambridge, US) compared with homologous vaccination composed of mRNA vaccines only (either Comirnaty and Spikevax) [1-4]. For example, binding and neutralising antibody titres were similar or greater in the heterologously boosted group compared with the homologous group [5,6]. In addition, we and others have reported that the enhanced immunogenicity of the heterologous vaccination regimen was associated with a better protection against severe acute respiratory syndrome coronavirus 2 (SARS-CoV-2) infection [2,7]. However, numerous studies have shown that the level of antibodies, and in particular those neutralising the virus, gradually decreases following vaccination. This phenomenon, combined with the emergence of viral variants having acquired mutations in the viral spike protein making them less sensitive to vaccine antibodies, led health authorities to recommend the injection of an additional booster dose. This booster dose was particularly important in immunocompromised patients for whom vaccine efficacy was lower. Heterologous vaccine schedules of Vaxzevria priming and mRNA booster doses as both second and third doses were not associated with increased risk of serious adverse events compared with homologous mRNA vaccine schedules [8]. 

Recent reports demonstrate that most COVID-19 vaccines delivered as a third dose booster significantly enhanced both humoral and cellular anti-SARS-CoV-2 immunity [9]. Observational studies also suggest that a third dose significantly improves protection from symptomatic infection compared with two doses. A recent meta-analysis reported that heterologous and homologous three-dose regimens work comparably well in preventing COVID-19 infections, even against different variants [10]. Nevertheless, a recent report has documented some differences in immunogenicity and protection according to vaccine schedule before third dose [11], suggesting that initial vaccination regimens could imprint spike-specific immunity in the long term, regardless of the booster dose.

To address this question, we compared spike-specific immunity and protection against infection conferred by a second and third dose of mRNA vaccine in healthcare workers (HCW) primed with either adenovirus-based Vaxzevria or a COVID-19 mRNA vaccine.

## Methods

### Prevalence of SARS-CoV-2 variants of concern

Testing of HCW for SARS-CoV-2 was performed using routine diagnostic procedures in the virology laboratory of the University Hospital of Lyon (Hospices Civils de Lyon, HCL) and included: transcription mediated amplification (TMA) (Aptima SARS-CoV-2 Assay, Hologic, Marlborough, US), loop-mediated isothermal amplification (LAMP) (SARS-CoV-2 ID NOW, Abbott, Sligo, Ireland) and RT-qPCR with different kits (Cobas 6800 SARS-CoV-2 assay, Roche, Basel, Switzerland or Panther Fusion SARS-CoV-2 assay, Hologic). To determine the prevalence of SARS-CoV-2 variants of concern (VOC) in HCW, available positive samples with quantification cycle (Cq) < 28 were sequenced using COVIDSeq (Illumina) as previously described [12]. Libraries were sequenced to 1 M paired-end reads (2 × 100 bp) and data were analysed using the in-house seqmet bioinformatic pipeline (available at https://github.com/genepii/seqmet). Clades and lineages were determined on samples with genome coverage > 90% using Nextclade and PangoLEARN, respectively.

### Cohort description

#### Population of HCW from the hospital database included in the epidemiological investigation

**Figure 1:**
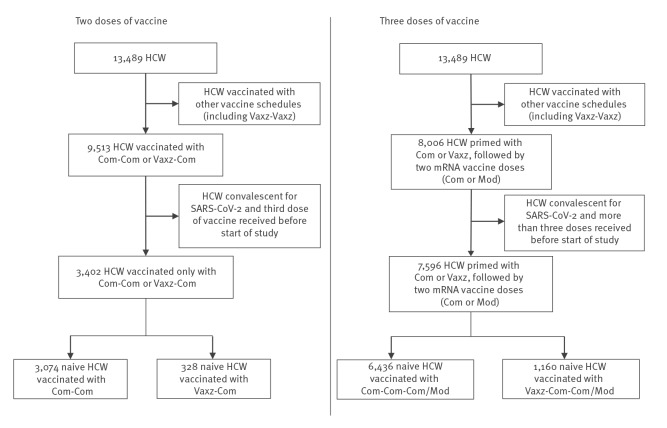
Selection of healthcare workers for the epidemiological study on COVID-19 booster vaccinations, Lyon, France, 15 December 2021–21 March 2022 (n = 13,489)

We extracted data from the occupational medicine database of the HCL. A total of 13,489 HCW working at HCL throughout the study period (15 December 2021–21 March 2022) were included. Only subjects who (i) had never contracted COVID-19, (ii) were primed with Vaxzevria or an mRNA vaccine and (iii) had received the second or third dose of an RNA vaccine were included in the epidemiological analysis. We provide the details of the recruitment in Figure 1. Breakthrough infections that were documented by positive RT-PCR or antigen tests and that occurred after 15 December 2021 and at least 7 days after vaccine injection were taken into account to evaluate infection risk in different groups of subjects. Because SARS-CoV-2 vaccination was mandatory for HCW in France, we have no missing data regarding this variable. Moreover, the declaration of SARS-CoV-2 infection was compulsory for all staff to obtain daily allowances without loss of salary during the imposed quarantine.

Com: Comirnaty; COVID-19: coronavirus disease; HCW: healthcare worker; Mod: Moderna; SARS-CoV-2: severe acute respiratory syndrome coronavirus 2; Vaxz: Vaxzevria.

The flowchart indicates how individuals with two or three vaccination doses were selected from the total HCW population according to the indicated criteria of exclusion.

#### Population included in the immune response investigations

Eighty-eight HCW naive for COVID-19 and vaccinated with Comirnaty and/or Vaxzevria and/or Spikevax were included in a prospective longitudinal cohort study conducted at the HCL. Blood sampling was performed before vaccination, before and 4 weeks after the second and the third dose of vaccine. The absence of previous SARS-CoV-2 infection was confirmed using the Abbott SARS-CoV-2 anti-N Ab total assay in all samples (Abbott Diagnostics, Abbott Park, Illinois, US).

### Measurement of IgG titres

Serum specimens were immediately stored at −80 °C after blood sampling. Receptor-binding domain (RBD)-specific IgG antibodies were measured using the Vidas SARS-CoV-2 IgG diagnostic kit (bioMérieux, Marcy-l'Étoile France). For standardisation of these assays to the first World Health Organization international standard, the concentrations were transformed into binding antibody units per mL (BAU/mL) using the conversion factors provided by the manufacturer.

### Live virus neutralisation experiments

A plaque reduction neutralisation test (PRNT) was used for the detection and titration of neutralising antibodies as previously described [13]. Neutralisation was recorded if more than 50% of the cells present in the well were preserved. Herein, the neutralising titre was expressed as the inverse of the highest serum dilution that exhibited neutralising activity; the detection threshold of 50% plaque reduction neutralisation (PRNT_50_) was ≥ 20 PRNT_50_ for neutralising antibodies. All experiments were performed with a subset of serum specimens collected longitudinally from 15 subjects in each group. The different viral strains that were used were sequenced and deposited in GISAID (https://www.gisaid.org/)

### Statistical analysis

The descriptive statistics generated appropriate figures and parameters according to type of variable (i.e. continuous or categorical). The comparisons between groups were done using the chi-squared test for categorical variables and non-parametric test or Student’s t-test according to the distribution for continuous variables. The cumulative probability of COVID-19 was based on the Kaplan–Meier survival analysis, and survival distributions were compared using the LogRank test. A univariate and multivariate Cox proportional hazard model was performed to identify the determinants independently associated with onset of COVID-19 according to their hazard ratio (HR) and their 95% confidence intervals (CI). All p values were two-tailed. A value of p < 0.05 was considered as statistically significant. To compare clinical parameters in the immunological study, Wilcoxon–Mann–Whitney two-sided tests were used for quantitative variables and chi-squared or Fisher’s exact test were used for qualitative variables when appropriate. Adjusted p values were calculated using the Benjamini–Hochberg method. To analyse biological data in the immunological study, we used a multiple linear regression model, with adjustment variables (age or age groups, sex, delay, vaccination scheme).

## Results

### Vaccine effectiveness in healthcare workers

Using the extracted database from the occupational medicine database of the HCL, we compared the risk of SARS-CoV-2 infection following a second or third dose of COVID-19 mRNA vaccine (Comirnaty or Spikevax) in subjects who received a priming dose of Vaxzevria or Comirnaty vaccine. As the third dose was mandatory for healthcare workers 6 months after the second dose, most of them had received their third dose before the start of the study, i.e. by 15 December 2021. This explains the lower proportion of HCW with two doses at the start of the study. We focused on individuals not previously infected with SARS-CoV-2 (PCR or antigenaemia-positive diagnosis) before vaccination to avoid any bias linked to hybrid immunity (Figure 1).

**Figure 2:**
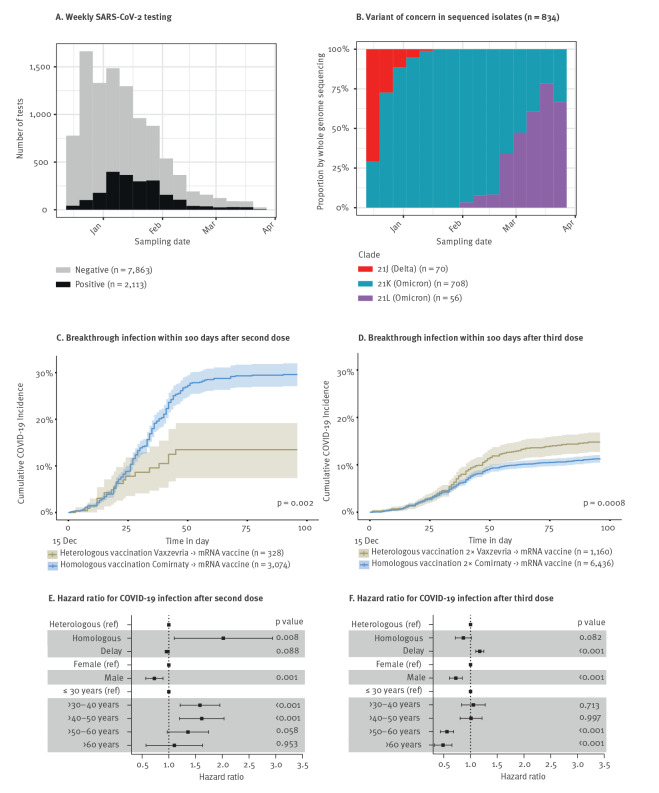
Effectiveness of heterologous vs homologous COVID-19 vaccination after the third vaccine dose, healthcare workers, Lyon, France, 15 December 2021–21 March 2022 (n = 13,489)

COVID-19: coronavirus disease; HR: hazard ratio; ref: reference; SARS-CoV-2: severe acute respiratory syndrome coronavirus 2.

We monitored infection rates in HCW after the second or the third booster dose in each group. We extracted SARS-CoV-2 infection incident events documented by positive antigen or RT-PCR tests that occurred between 15 December 2021 and 21 March 2022. This period corresponded to the Omicron (lineage BA.1, Clade 21K) wave, succeeding the Delta variant (lineage B.1.1.529, Clades 21I and 21J) and preceding the appearance and increase of the Omicron sub-lineage BA.2 (Clade 21L) (Figure 2A-B). The proportion of SARS-CoV-2 VOC determined by whole genome sequencing in 834 samples collected in HCW working at HCL during this period was: 70 (8.4%) 21J (Delta), 708 (84.9%) 21K (Omicron BA.1) and 56 (6.7%) 21L (Omicron BA.2). Figure 2C-D shows the cumulative incidence of breakthrough infections in each group. Following the second vaccine dose, heterologous vaccination regimens were more protective against infection compared with the homologous regimen group (Figure 2C). Indeed, after adjustment for age, sex and delay between last vaccination and the start of the study, individuals vaccinated with two doses of mRNA vaccines were twice as likely to be infected than those vaccinated with Vaxzevria followed by mRNA vaccine (adjusted HR = 1.88; 95% CI: 1.18–3.00; p = 0.008) (Figure 2E). After the third dose, the number of infections was lower than after the second dose in both vaccination groups, showing the benefit of the booster dose. Moreover, in the homologous group, the third dose achieved at least the same level of protection as in the heterologous group as demonstrated by the inversion of the infection incidence curves (adjusted HR = 0.86; 95% CI: 0.72–1.02; p = 0.082) (Figure 2F). We also analysed the infection risk according to the parameters of vaccination, sex or age class. This analysis confirmed the importance of the vaccination regimen after the second dose but not after the third (Figure 2E-F). It also shows that in this cohort of vaccinated HCW, middle-aged female HCW had a higher risk of infection.

Panels C and D: p value was calculated using Logrank test. The shaded areas show 95% confidence intervals. Panels E and F: Forest plots of multivariate Cox proportional HR with 95% confidence intervals (whiskers), calculated with the HR of 1.0 of the reference stratum. All SARS-CoV-2 infection events documented by positive SARS-CoV-2 RT-PCR or antigen test were recorded by the service of occupational medicine, Hospices Civils de Lyon.

### Analysis of immune response after heterologous and homologous vaccination

**Figure 3:**
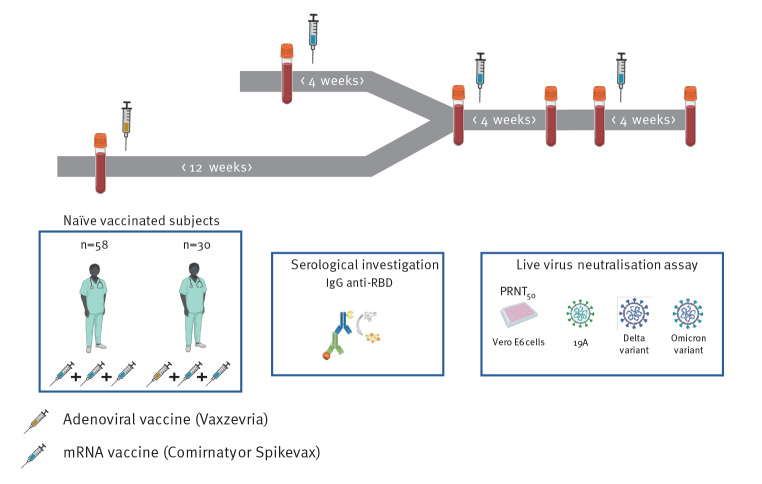
Design of the *Covid-Ser* study, France January 2021–February 2022 (n = 88)

PRNT50: 50% plaque reduction neutralisation test; RBD: Receptor binding domain.

We sought to compare the immunogenicity of the third dose in heterologous vs homologous vaccination groups. For this, we took advantage of the *Covid-Ser* cohort that we have previously described and which includes a subset of voluntary HCW in whom anti-SARS-CoV-2 immunity is measured longitudinally over time (Figure 3) [2]. We formed groups who had received a homologous or heterologous vaccination regimen and who had received a third dose of mRNA vaccine; their demographic characteristics and delays between doses are shown in the Table.

**Table:** Clinical characteristics of patients in the *Covid-Ser* study (immunological analysis), France January 2021–February 2022 (n = 88)

	HCW primed with Comirnaty (homologous) n = 58	HCW primed with Vaxzevria (heterologous) n = 30	p adjusted
Male sex	11	6	> 0.9999
Female sex	47	24	> 0.9999
Median age in years (IQR)	50.50 (41.00–59.00)	38.00 (30.50–41.00)	< 0.0001
Median body mass index^a^ (IQR)	24.22 (22.12–29.72)	20.94 (20.06–23.88)	0.0077
Median delay between first and second dose in days (IQR)	28 (28–30)	85 (84–85)	< 0.0001
Median delay between second and third dose in days (IQR)	266 (245–284)	206 (195–213)	< 0.0001
Third dose Spikevax vaccine	5	3	> 0.9999
Presence of comorbidity	29	10	0.5828
Description of comorbidities			
Hypertension	2	0	> 0.9999
Diabetes	2	0	> 0.9999
Cancer	1	0	> 0.9999
Hypothyroidism	4	1	> 0.9999
Rheumatic diseases	1	0	> 0.9999
Chronic respiratory problems	4	0	0.7200
Other comorbidities	5	0	0.5480
Currently smoker	14	6	> 0.9999

IQR: interquartile range; HCW: healthcare worker.

^a^ There were missing data in this category, we recorded this information for 55/58 and 29/30 in homologous and heterologous groups, respectively.

^b^ Consumption at least once a week.

**Figure 4:**
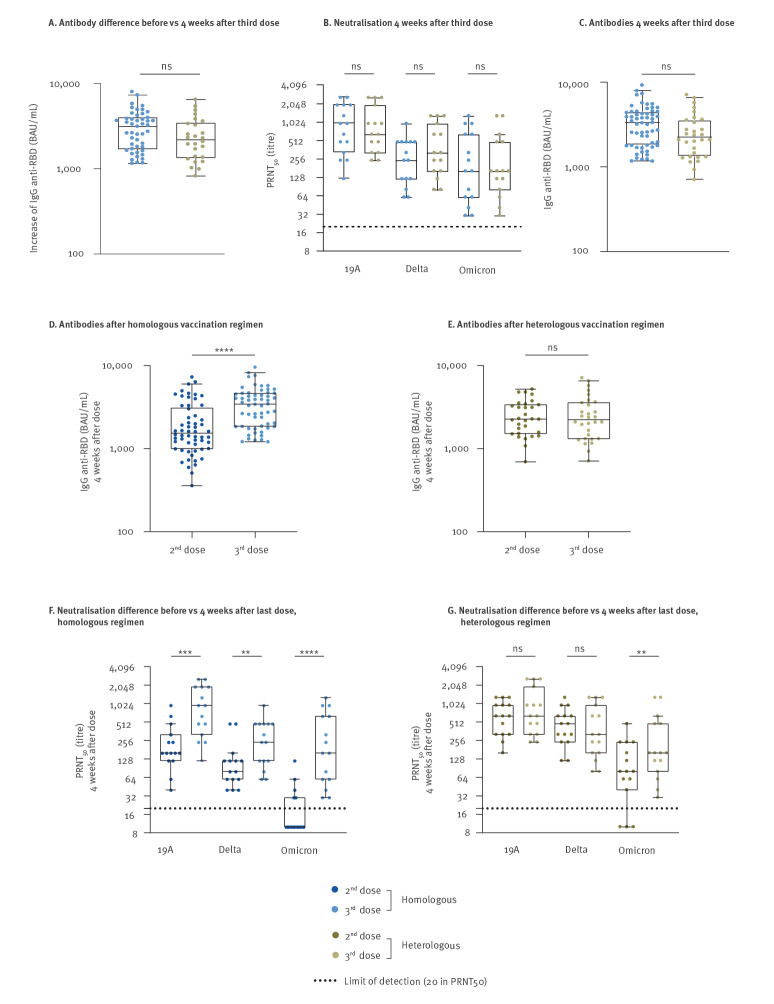
The booster dose equalises the levels of immunogenicity of heterologous or homologous vaccine regimens, France, January 2021–February 2022 (n = 88)

BAU: binding antibody units; ns: not significant (p value > 0.05); PRNT: plaque reduction neutralisation test; RBD: receptor-binding domain.

Subsequently, we measured the increase in anti-RBD IgG antibody levels 4 weeks after the third dose in both groups. No significant difference was observed (p = 0.14) (Figure 4A). We then evaluated the neutralisation capacity of serum antibodies against SARS-CoV-2 variants 19A, Delta and Omicron 4 weeks after the third dose. No significant difference in neutralisation was observed between the homologous and heterologous schedules (19A: median: 960 (IQR: 320–1,920) vs 640 (IQR: 320–1,920); Delta: 240 (IQR: 120–480) vs 320 (IQR: 160–960); Omicron: 160 (IQR: 60–640) vs 160 (IQR: 80–480), respectively) (Figure 4B). Moreover, the anti-RBD IgG level was not different in the two groups after the third dose (p = 0.18) (Figure 4C).

** p < 0.01; *** p < 0,001; **** p < 0,0001.

Sera from naïve HCW vaccinated primed with the Comirnaty (n = 58) or Vaxzevria vaccine, n = 30) and who received an mRNA vaccine for the second and third dose were selected. Panel A: The pre-vaccination time point was missing for 11 and 4 patients vaccinated with a homologous and heterologous regimen, respectively. Panels B, F and G: A subset of 15 sera from each group were assayed in duplicate for their capacity to neutralise infection by different SARS-CoV-2 strains in Vero E6 cells.

Data are represented as box-and-whiskers plots using the Tukey method which show median (horizontal line inside the box) and interquartile range (25–75%: upper and lower horizontal lines of the box), and each dot corresponds to one healthcare worker. Statistics were calculated using a multiple linear regression model with adjustment variables (age and sex, and when appropriate delay between second and third dose).

In Figure 2, the heterologous Vaxzevria/COVID-19 mRNA vaccine combination conferred better protection against SARS-COV-2 infection than the homologous COVID-19 mRNA vaccines combination, but the third dose equalised the efficacy of both vaccine regimens. To understand why the advantage of heterologous vaccination was no longer observed after the third dose, we compared humoral immunity 4 weeks after the second and after the third dose in each group. In subjects vaccinated according to the homologous regimen (Figure 4D), the anti-RBD IgG level measured 4 weeks post second dose was significantly lower (p < 0.0001) than that measured post third dose (1,490 BAU/mL (95% CI: 969–2,994) vs 3,336 BAU/mL (95% CI: 1,795–4,491)), whereas in subjects vaccinated according to a heterologous scheme (Figure 4E), we observed no difference (2,277 BAU/mL (95% CI: 1,520–3,400) vs 2,242 BAU/mL (95% CI: 1,321–3,602)). In addition, for the homologous vaccine group, the antibody neutralising capacity 4 weeks post third dose was at least three times higher than that observed 4 weeks post second dose (p < 0.01 or lower). In contrast, in heterologously vaccinated individuals, the third dose conferred no benefit in neutralising capacity against the 19A and Delta variants at 4 weeks. Only the neutralising capacity of the total antibodies against Omicron increased twofold 4 weeks after the third compared with 4 weeks after the second dose (p < 0.01) (Figure 4F-G). Of note, 4 weeks after the third dose there was no difference in the anti-SARS-CoV-2 IgG level between HCW boosted with Comirnaty (n = 80) or Spikevax (n = 8) (p = 0.30).

## Discussion

We previously showed in a real-world observational cohort study in HCW that the heterologous Vaxzevria/Comirnaty combination confers better protection against SARS-CoV-2 infection than the homologous Comirnaty/Comirnaty combination [2]. Both combinations induced strong anti-spike antibody responses but serum specimens from heterologously vaccinated individuals displayed stronger neutralising activity, regardless of the SARS-CoV-2 variant [14]. Here, we asked whether the advantage conferred by the heterologous regimen is conserved after a booster dose with an mRNA-based COVID-19 vaccine. 

Our results show that (i) the third dose with an mRNA vaccine equalises the levels of effectiveness of heterologous or homologous COVID-19 vaccine regimens and (ii) that serum neutralisation capacity against different SARS-CoV-2 variants is comparable in both groups 4 weeks after the booster. Indeed, the third vaccine dose did not increase antibody levels and neutralisation capacity beyond those observed 1 month after the second dose in the heterologous group, which suggests that a maximal immunity level 1 month post vaccination is already reached after the second dose in this group and cannot be boosted further, at least with an mRNA-based vaccine. While we cannot exclude that either a shorter or a longer delay between the second and third doses would have a different impact in the heterologous vs homologous group, our results are in line with those of Accorsi et al. who showed that a single booster dose of an mRNA Covid-19 vaccine in individuals who had received primary vaccination with a single dose of the adenovirus-based COVID-19 Vaccine Janssen (Ad26.COV2.S, Janssen-Cilag International NV, Beerse, Belgium), provided protection close to that of the three-dose mRNA vaccine regimen [15]. In addition, Behrens et al. reported that inferior SARS-CoV-2-specific immune responses following homologous Vaxzevria/Vaxzevria vaccination compared with Vaxzevria/Comirnaty can be compensated by heterologous Comirnaty vaccination as the third dose [16]. In our study, only the neutralisation of the Omicron variant was slightly better 1 month after the third dose in the heterologous regimen compared with the second dose. The significance of this result is not clear since the neutralisation of the other variants was not changed.

Our results address the question of how many vaccine booster doses are needed to reach maximal humoral immunity in both anti-RBD IgG level and neutralisation capacity against SARS-CoV-2, including its numerous variants. We confirm that, using the heterologous combination performed in our study, a single boost was shown to be enough in our study to reach this plateau. By contrast, the homologous scheme using an mRNA vaccine needs two boosters for reaching the same level of protection. Of course, this maximum immunity is temporary and decreases with time, which makes the third dose necessary in the heterologous group as well. The recent study of Regev-Yochay et al. that evaluated the benefit of a third booster in a homologous mRNA immunisation scheme suggests that maximum immunity is reached after the third dose with homologous mRNA vaccination. The fourth mRNA vaccine dose seems to be able to restore the level of immunity after waning but does not quantitatively and qualitatively improve the humoral immunity conferred by the first three doses [17].

Surprisingly, our data highlighted a higher risk of infection in women between 30 and 50 years compared with older HCW. This group of individuals mainly corresponds to active nursing staff with higher exposure to pathogens. Indeed, at the HCL, contacts between nurses and patients and among nurses are longer and more frequent than for other professionals [18], and this pattern could result in a higher exposure to pathogens, as previously reported in a study of nosocomial influenza spreading in hospitals [19]. Obviously, this higher exposure of middle-aged women is specific to the HCW community and may not apply to the general population.

Some limitations of the present study should be acknowledged. Firstly, these results were obtained from observational data and not from a randomised clinical trial. As a result, there were some inherent differences between the compared groups. For example, subjects vaccinated with a heterologous schedule were on average younger than those vaccinated with a homologous schedule. This difference is explained by the recommendations for vaccination according to which individuals under 55 years of age who received a first dose of Vaxzevria should receive a booster of COVID-19 mRNA vaccine. Yet, our statistical analysis did not show a significant impact of age in infection risk or vaccine-induced immune parameters. Secondly, the HCW might differ slightly from the general population since they are repeatedly exposed to SARS-CoV-2 and closely monitored for vaccine coverage and COVID-19 incidence. Thirdly, in our study, the advantage of the heterologous regimen observed after the second dose may be impacted by the delay between the first and second dose which was only 4 weeks between the first two doses in the homologous scheme. A study by Payne et al. reported that an extended delay of 10 weeks between the first two doses of Comirnaty allowed the development of better humoral immunity [20]. Fourthly, the present study was limited to HCW without history of SARS-CoV-2 infection before vaccination. The benefit of an mRNA vaccine booster dose in patients primed with Vaxzevria or mRNA vaccine would need further investigation in people previously infected by different SARS-CoV-2 variants. Finally, even if HCW were heavily tested during the monitoring period of our study, asymptomatic infections may have remained unnoticed.

## Conclusion

Our data provide evidence to understand the number of vaccine booster doses needed to reach the maximal level of both antibody titre and neutralisation capacity against SARS-CoV-2 in heterologous or homologous vaccine scheme. More studies will be needed to determine if another vaccine type should be given to boost SARS-CoV-2 immunity even further.

## References

[1] Schmidt T Klemis V Schub D Mihm J Hielscher F Marx S Immunogenicity and reactogenicity of heterologous ChAdOx1 nCoV-19/mRNA vaccination. Nat Med. 2021;27(9):1530-5. 10.1038/s41591-021-01464-w 34312554 PMC8440177

[2] Pozzetto B Legros V Djebali S Barateau V Guibert N Villard M Immunogenicity and efficacy of heterologous ChAdOx1-BNT162b2 vaccination. Nature. 2021;600(7890):701-6. 10.1038/s41586-021-04120-y 34673755

[3] Liu X Shaw RH Stuart ASV Greenland M Aley PK Andrews NJ Safety and immunogenicity of heterologous versus homologous prime-boost schedules with an adenoviral vectored and mRNA COVID-19 vaccine (Com-COV): a single-blind, randomised, non-inferiority trial. Lancet. 2021;398(10303):856-69. 10.1016/S0140-6736(21)01694-9 34370971 PMC8346248

[4] Klemis V Schmidt T Schub D Mihm J Marx S Abu-Omar A Comparative immunogenicity and reactogenicity of heterologous ChAdOx1-nCoV-19-priming and BNT162b2 or mRNA-1273-boosting with homologous COVID-19 vaccine regimens. Nat Commun. 2022;13(1):4710. 10.1038/s41467-022-32321-0 35953492 PMC9366133

[5] Hillus D Schwarz T Tober-Lau P Vanshylla K Hastor H Thibeault C Safety, reactogenicity, and immunogenicity of homologous and heterologous prime-boost immunisation with ChAdOx1 nCoV-19 and BNT162b2: a prospective cohort study. Lancet Respir Med. 2021;9(11):1255-65. 10.1016/S2213-2600(21)00357-X 34391547 PMC8360702

[6] Niyomnaitham S Quan Toh Z Wongprompitak P Jansarikit L Srisutthisamphan K Sapsutthipas S Immunogenicity and reactogenicity against the SARS-CoV-2 variants following heterologous primary series involving CoronaVac, ChAdox1 nCov-19 and BNT162b2 plus BNT162b2 booster vaccination: An open-label randomized study in healthy Thai adults. Hum Vaccin Immunother. 2022;18(6):2091865. 10.1080/21645515.2022.2091865 35816053 PMC9746495

[7] Mayr FB Talisa VB Shaikh O Yende S Butt AA . Effectiveness of homologous or heterologous Covid-19 boosters in veterans. N Engl J Med. 2022;386(14):1375-7. 10.1056/NEJMc2200415 35139265 PMC8849183

[8] Andersson NW Thiesson EM Laursen MV Mogensen SH Kjær J Hviid A . Safety of heterologous primary and booster schedules with ChAdOx1-S and BNT162b2 or mRNA-1273 vaccines: nationwide cohort study. BMJ. 2022;378:e070483. 10.1136/bmj-2022-070483 35831006 PMC9277486

[9] Lustig Y Gonen T Meltzer L Gilboa M Indenbaum V Cohen C Superior immunogenicity and effectiveness of the third compared to the second BNT162b2 vaccine dose. Nat Immunol. 2022;23(6):940-6. 10.1038/s41590-022-01212-3 35534723

[10] Au WY Cheung PPH . Effectiveness of heterologous and homologous covid-19 vaccine regimens: living systematic review with network meta-analysis. BMJ. 2022;377:e069989. 10.1136/bmj-2022-069989 35640925 PMC9724446

[11] Liu X Munro APS Feng S Janani L Aley PK Babbage G Persistence of immunogenicity after seven COVID-19 vaccines given as third dose boosters following two doses of ChAdOx1 nCov-19 or BNT162b2 in the UK: Three month analyses of the COV-BOOST trial. J Infect. 2022;84(6):795-813. 10.1016/j.jinf.2022.04.018 35405168 PMC8993491

[12] Bal A Simon B Destras G Chalvignac R Semanas Q Oblette A Detection and prevalence of SARS-CoV-2 co-infections during the Omicron variant circulation in France. Nat Commun. 2022;13(1):6316. 10.1038/s41467-022-33910-9 36274062 PMC9588762

[13] Saade C Gonzalez C Bal A Valette M Saker K Lina B Live virus neutralization testing in convalescent patients and subjects vaccinated against 19A, 20B, 20I/501Y.V1 and 20H/501Y.V2 isolates of SARS-CoV-2. Emerg Microbes Infect. 2021;10(1):1499-502. 10.1080/22221751.2021.1945423 34176436 PMC8330769

[14] Lee HK Go J Sung H Kim SW Walter M Knabl L Heterologous ChAdOx1-BNT162b2 vaccination in Korean cohort induces robust immune and antibody responses that includes Omicron. iScience. 2022;25(6):104473. 10.1016/j.isci.2022.104473 35637788 PMC9132682

[15] Accorsi EK Britton A Shang N Fleming-Dutra KE Link-Gelles R Smith ZR Effectiveness of Homologous and Heterologous Covid-19 Boosters against Omicron. N Engl J Med. 2022;386(25):2433-5. 10.1056/NEJMc2203165 35613039 PMC9165559

[16] Behrens GMN Barros-Martins J Cossmann A Ramos GM Stankov MV Odak I BNT162b2-boosted immune responses six months after heterologous or homologous ChAdOx1nCoV-19/BNT162b2 vaccination against COVID-19. Nat Commun. 2022;13(1):4872. 10.1038/s41467-022-32527-2 35982040 PMC9387891

[17] Regev-Yochay G Gonen T Gilboa M Mandelboim M Indenbaum V Amit S Efficacy of a fourth dose of Covid-19 mRNA vaccine against Omicron. N Engl J Med. 2022;386(14):1377-80. 10.1056/NEJMc2202542 35297591 PMC9006792

[18] Vanhems P Barrat A Cattuto C Pinton JF Khanafer N Régis C Estimating potential infection transmission routes in hospital wards using wearable proximity sensors. PLoS One. 2013;8(9):e73970. 10.1371/journal.pone.0073970 24040129 PMC3770639

[19] Voirin N Payet C Barrat A Cattuto C Khanafer N Régis C Combining high-resolution contact data with virological data to investigate influenza transmission in a tertiary care hospital. Infect Control Hosp Epidemiol. 2015;36(3):254-60. 10.1017/ice.2014.53 25695165

[20] Payne RP Longet S Austin JA Skelly DT Dejnirattisai W Adele S Immunogenicity of standard and extended dosing intervals of BNT162b2 mRNA vaccine. Cell. 2021;184(23):5699-5714.e11. 10.1016/j.cell.2021.10.011 34735795 PMC8519781

